# Reducing Tobacco Use in Oregon Through Multisector Collaboration: Aligning Medicaid and Public Health Programs

**DOI:** 10.5888/pcd17.200220

**Published:** 2020-12-10

**Authors:** Catherine J. Livingston, Sarah E. Bartelmann, Nancy M. Goff, Kirsten G. Aird

**Affiliations:** 1 Oregon Health & Science University, Portland, Oregon; 2 Oregon Health Authority, Health Policy and Analytics, Portland, Oregon; 3 Oregon Health Authority, Public Health Division, Portland, Oregon

## Abstract

**Introduction:**

Tobacco use is the leading cause of preventable death and disease in the United States. Oregon’s coordinated care model for Medicaid provides an opportunity to consider novel ways to reduce tobacco use.

**Purpose and Objectives:**

We sought to evaluate the changes in tobacco cessation benefits, patient access to cessation interventions, and cigarette smoking prevalence before and after introduction of the statewide Coordinated Care Organization (CCO) cigarette smoking incentive metric for Medicaid members.

**Intervention Approach:**

Medicaid and public health collaborated to develop a novel population-level opportunity to reduce tobacco use. In 2016, an incentive metric for cigarette smoking was incorporated into Oregon’s CCO Quality Incentive Program, which holds Oregon’s CCOs accountable for providing comprehensive cessation benefits and for reducing tobacco use prevalence among members.

**Evaluation Methods:**

We evaluated the changes in tobacco cessation benefits, patient–provider discussions of smoking cessation, and cigarette smoking prevalence before and after the introduction of the statewide CCO cigarette smoking incentive metric.

**Results:**

All 15 CCOs now cover cessation counseling (telephone, individual, and group) and pharmacotherapy (all 7 FDA-approved medications). The number of CCOs requiring prior authorization for at least 1 FDA-approved pharmacotherapy decreased substantially. From 2016 through 2018, the percentage of Medicaid members who reported that their health care providers recommended cessation assistance increased above baseline. The incentive metric and aligned interventions were associated with a reduction in cigarette smoking prevalence among Medicaid members, as indicated by the electronic health record metric. Thirteen of 15 CCOs demonstrated a reduction in smoking prevalence with the statewide prevalence rate decreased from 29.3% to 26.6%.

**Implications for Public Health:**

Since incentive metric implementation, progress has been made to reduce tobacco use among CCO members. Cross-agency partnerships between Medicaid and public health contributed to these successes.

**Summary**

**What is known on this topic?**

Tobacco use can be reduced with evidence-based cessation strategies such as improving access to cessation counseling and medications as well as community-based interventions.

**What is added by this report?**

We describe the efforts of a state health agency to improve access to cessation benefits and reduce tobacco use through the creation and implementation of a novel incentive metric for Oregon’s Medicaid delivery organizations.

**What are the implications for public health practice?**

Medicaid and public health agencies can work together to reduce tobacco use through policy and systems levers both inside and outside of clinics.

## Introduction

Tobacco use is the leading cause of preventable illness and death in the United States. In 2018, 19.7% of people used any tobacco product, and use was disproportionately higher among those who have Medicaid (27.8%) (1). In Oregon, tobacco use is associated with more than 8,000 deaths each year (2) and costs Oregon $2.9 billion annually in medical expenditures, lost productivity, and premature death (3). The negative effects of tobacco are most damaging to low-income Oregonians, members of certain racial and ethnic groups, tribal members, members of the LGBTQ community, and people with mental illness, all of whom use tobacco at higher rates than their counterparts and have the most severe health consequences as a result (4). Data from Oregon’s 2017 Behavioral Risk Factor Surveillance System (BRFSS) indicate that the prevalence of tobacco use is higher among Oregon adults enrolled in Medicaid: 27% of Oregon adults enrolled in Medicaid smoke cigarettes, compared with 15% of those with other types of insurance (4) (Box).

**Box. Cigarette Smoking and Tobacco Use Definitions**

The term “cigarette smoking” in this article is used to describe the process of inhaling tobacco smoke from combustible cigarettes. “Tobacco use” is broader and generally includes the use of cigars, e-cigarettes, smokeless tobacco, and other tobacco and vaping products.

Reducing tobacco use requires a multifaceted approach that prevents youth and young adults from initiating tobacco use, eliminates tobacco-related health disparities in all populations, minimizes exposure to secondhand smoke, and helps tobacco users quit (6). Key elements of this approach include promoting and improving access to affordable and effective cessation services, as well as ensuring that the places people live, work, play, and learn are tobacco-free and reinforce individuals’ desire to quit or never start using tobacco.

The evidence-based clinical strategies that underpin this comprehensive approach, along with evidence of successful cross-sector collaboration between public health and health care, are well documented in the literature (7–9). The US Preventive Services Task Force (USPSTF) conducts systematic reviews and has identified evidence-based clinical interventions to reduce tobacco use. The USPSTF gives a grade “A” to the recommendation (defined as a recommended service because of high certainty that the net benefit is substantial) that clinicians ask all adults about tobacco use, advise users to quit, and provide behavioral counseling interventions and pharmacotherapy approved by the US Food and Drug Administration (FDA) (10). Furthermore, the Centers for Disease Control and Prevention identified 3 key evidence-based health systems interventions that are proven to accelerate tobacco use reduction — increasing access to cessation counseling and medications, removing barriers to access such as copays or coinsurance, and promoting the increased use of cessation benefits by tobacco users (11).

Oregon’s creation of coordinated care organizations (CCOs) in 2012 (12) provided an unprecedented opportunity to reduce tobacco use among Medicaid members through implementation of the evidence-based systems and policy changes just described. CCOs are health care plans that coordinate health care delivery for the Oregon Health Plan (OHP, Oregon’s Medicaid program). CCOs were introduced as a key component of Oregon’s coordinated care model with the goal of transforming Oregon’s health system to provide incentives to better health and better care at lower costs. As part of the CCO Quality Incentive Program, CCOs are required to report annually on quality improvement and health outcome metrics, hereafter referred to as *incentive metrics*, for which they receive payment for performance if they meet certain benchmarks or targets (13). A variety of existing tobacco measures were considered for adoption, but none met the level of desired population impact. Existing tobacco measures (eg, through the National Quality Forum) (14) hold no accountability for the outcome of decreasing tobacco use. Instead, these measures focus on screening individual patients for tobacco use (a process measure alone), or a step up, measure whether a patient received an intervention of counseling, pharmacotherapy, or both. In developing an innovative Medicaid payer metric with a population health lens, the critical areas identified as necessary to an effective measure included coverage of the suite of evidence-based interventions, including minimum standards of pharmacotherapy and counseling, and accountability for reducing tobacco use prevalence (ie, ensuring that the interventions were actually effective and resulted in a decrease in smoking rates, arguably a more patient-oriented outcome). Holding plans, rather than providers alone, accountable may increase the effectiveness of the metric on population health outcomes. The use of substantial financial incentives also promotes investment in achieving the metric. During development of this program, we were not aware of any other states looking into holding health plans accountable for tobacco use prevalence or using financial incentives for this work, making this a highly innovative approach. Although additional developments on national tobacco measures that could be used for prevalence have occurred, we are unaware of any state programs using these measures for health plan accountability or to provide incentives for performance.

In 2016, a novel incentive metric for cigarette smoking was implemented, holding CCOs accountable for providing comprehensive cessation benefits as well as reducing tobacco use prevalence among members. As a result, public health, Medicaid, and the CCOs are aligned with the common goal of reducing tobacco use in Oregon.

## Purpose and Objective

We sought to evaluate the changes in tobacco cessation benefits, patient access, and cigarette smoking prevalence before and after the introduction of the statewide CCO cigarette smoking incentive metric for Medicaid members in 2016.

We describe 1) the history of related tobacco efforts that led to the creation of the incentive metric; 2) the current state and local policy and system infrastructure that supports CCO success on tobacco use reduction; and 3) the impact of these efforts on tobacco cessation benefits and cigarette smoking rates for Oregon’s Medicaid population.

## Intervention Approach

### Oregon’s history of cross-agency collaboration on tobacco reduction

The introduction of the cigarette smoking incentive metric is grounded in a history of collaboration between public health and health care partners throughout the Oregon Health Authority (OHA). The Tobacco Prevention and Education Program (TPEP), housed in the Public Health Division, began operating in 1997 with the passage of Measure 44 (15), which increased the price of tobacco in Oregon and dedicated a portion of the tobacco taxes to tobacco prevention and education (16). In 1997, the OHP (Medicaid) Prioritized List of Health Services added coverage for tobacco cessation services (17). In 1998, Oregon became one of the first states to offer free cessation services to all people in Oregon through the statewide tobacco quitline (18). Given the support for addressing tobacco use comprehensively (through taxes, Medicaid coverage, and public health), both TPEP and Medicaid provided staff resources to jointly implement a series of performance improvement projects with the goal of incentivizing the Medicaid managed care plans to promote tobacco cessation benefits to pregnant women, adolescents, and clients with chronic diseases, such as asthma, diabetes, and cardiovascular disease (19). When CCOs were established in 2012, public health and Medicaid staff were ready to work together to identify a common set of benefit design recommendations for tobacco cessation. These early cross-agency initiatives provided the foundation for a sustained and robust partnership across OHA to reduce tobacco use.

### Oregon’s tobacco prevention and cessation infrastructure

OHA currently leads tobacco reduction initiatives across multiple divisions that are responsible for both Medicaid and statewide public health outcomes. Since the inception of the TPEP program in 1997, cigarette smoking has decreased by more than 50% (4). TPEP’s work contributed to this success through implementation of evidence-based policy strategies such as increasing smoke-free environments in collaboration with local public health partners and tribes, increasing public awareness about the dangers of tobacco use through statewide education and advertising campaigns, and supporting access to cessation services through the statewide quitline. TPEP also maintains a robust tobacco surveillance and evaluation system to track, measure, and analyze tobacco-related data, and to use findings to inform program and policy approaches (20).

The Medicaid program also has taken a comprehensive approach to reduce tobacco use among Medicaid members. In Oregon, the state legislature determines the Medicaid benefit package by drawing a funding line on the state’s Prioritized List of Health Services, with services “above the line” being covered and those “below the line” not being covered. The Health Evidence Review Commission (HERC) is a governor-appointed commission representing physicians, community members, and CCOs that manages Oregon’s unique Prioritized List, which emphasizes covering services that are evidence-based, maximize population health, and control costs. HERC has assigned a very high priority to tobacco cessation and since 2016 requires that all CCOs offer Medicaid patients the “gold standard” evidence-based cessation interventions, including FDA-approved pharmacotherapy and behavioral counseling (21).

The Metrics and Scoring Committee, another statewide governor-appointed committee, is responsible for identifying incentive metrics for the CCO Quality Incentive Program (22). Performance metrics are increasingly used to promote change in health systems, clinical practice, and payer strategies and to create accountability (23). Although health plans frequently use process metrics such as whether members are offered tobacco cessation counseling services, health plans are generally not held accountable for outcome metrics such as tobacco use prevalence in their member population. The Metrics and Scoring Committee has driven innovation and performance improvements in Oregon by expanding CCO incentive measures to include outcome metrics such as the tobacco use reduction outcome measure.

### Introduction of the tobacco incentive metric

CCO incentive metrics are selected each year by the Metrics and Scoring Committee. Throughout the year, the Committee considers proposals for new incentive metrics from various sources, including public testimony from community members, presentations from state agency staff and subject matter experts, recommendations from its Technical Advisory Workgroup, and in some years, a widely distributed stakeholder survey. The committee has adopted incentive metric selection and retirement criteria that it uses to determine which (and when) new metrics should be added or existing metrics removed from the CCO incentive metric set. Several key criteria that the committee considers include alignment with other metric sets and consistency with other state priorities.

A tobacco prevalence metric was proposed for inclusion in the CCO incentive metric set soon after the establishment of CCOs in 2012; however, the only potential data source for a health plan–level, Medicaid-specific prevalence metric was the annual Consumer Assessment of Healthcare Providers and Systems (CAHPS) survey, and stakeholders expressed concerns that the survey sample was insufficient to fairly capture quality improvement efforts by health plans to reduce prevalence. Throughout 2014 and 2015, potential incentive metric options — both process and outcome measures — were discussed. Before the committee approved a metric for the 2016 performance year, public health advocates provided expert testimony on the importance of focusing on tobacco use prevalence. The release of Oregon’s State Health Improvement Plan in 2015 identified the prevention and reduction of tobacco use as a top priority, which also helped to elevate and sustain interest in the issue.

After the tobacco incentive metric was approved, the technical advisory workgroup developed the metric specifications based on Meaningful Use standards required for electronic health records (EHRs) and HERC requirements for tobacco cessation benefits.

### Supporting CCOs for success in reducing tobacco prevalence

Since the introduction of the incentive metric in 2016, various OHA divisions and committees have been working together to ensure alignment across state-level programs, policies, and systems and to support CCOs in their efforts to reduce tobacco prevalence.

**Cross-agency alignment.** Concurrent with the introduction of the incentive metric, HERC modified the Prioritized List of Health Services in 2015 to clarify the CCO requirement to cover effective clinical strategies, including behavioral health interventions (eg, quitlines, clinical counseling) and FDA-approved pharmacotherapy. Public health and HERC also worked to ensure that comprehensive, gold-standard cessation benefits (as defined by both HERC and the 2010 Patient Protection and Affordable Care Act) (24) were aligned across the Prioritized List, the public health guidance documents, and the surveys that assess CCO performance on the metric. In addition, the OHA Transformation Center works in partnership with public health to provide technical assistance and training to CCOs and providers on best practices to prevent and reduce tobacco use (25). This service includes clinical provider trainings, technical assistance on policy strategies, and trainings on how to use quitline data.

**Multisector Intervention Statements.** Several years ago, HERC developed a concept of Multisector Intervention Statements to address the fact that improvement in health outcomes may sometimes be more efficiently achieved by using strategies that occur outside of a clinician–patient in-person visit (26). The idea is to apply the same evidence standards as those for traditional clinical interventions so that health care plans can invest in the most effective evidence-based interventions to improve health outcomes, even if they are outside of the traditional health care setting.

In 2016, HERC reviewed high-quality systematic reviews and compiled the information in a summary of effective community-level interventions for tobacco use prevention and reduction plus a specific evidence evaluation for tobacco use during pregnancy. Using the findings of these reviews, HERC issued a multisector intervention statement (27,28) on tobacco use that outlines effective evidence-based interventions targeted at the community or population level, such as tobacco taxes and smoke-free laws. The goal of the multisector intervention statement is to provide CCOs the information they need to reduce cigarette smoking prevalence in their memberships and larger communities and to encourage them to play a role in implementing evidence-based community-level strategies alongside their local public health counterparts.

**Connecting local public health departments and CCOs.** OHA has also worked to connect the statewide network of TPEP in all counties and tribes with their regional CCOs to replicate the comprehensive scope of tobacco use prevention and cessation activities at the local level. Often TPEP provides data and implement policy and systems change strategies, while CCOs implement clinical improvement strategies. The introduction of a cigarette smoking incentive metric focused on prevalence reduction presents an opportunity for local public health and CCOs to form strategic partnerships to implement strategies that work. The Sustainable Relationships for Community Health grant program administered by the OHA Public Health Division serves to accelerate these local cross-sectoral partnerships by bringing CCO, local public health, clinical, and community-based partners together several times a year to work together on large-scale systems changes to reduce and prevent tobacco use. The OHA Public Health Division also works to connect local TPEP with other local partners, like maternal and child health programs that prioritize cessation in pregnant women, and alcohol and other drug prevention programs that address similar addiction issues.

## Evaluation Methods

The incentive metric for cigarette smoking prevalence was designed to have multiple components and to be phased in slowly to ensure that CCOs were meeting minimum cessation benefit requirements and that they and their contracted providers had sufficient infrastructure to support reporting on cigarette smoking prevalence from EHRs before being accountable for reductions in smoking prevalence. With these design parameters in mind, the resulting incentive metric has 3 components: 1) providing the minimum cessation benefit package, as defined by HERC, 2) reporting of EHR-based prevalence data, and 3) reducing cigarette smoking prevalence among CCO members (29).

Each component of the cigarette smoking incentive metric is worth a certain percentage of the total metric calculation, and each year CCOs must meet a certain overall performance target to earn incentive dollars. The component percentages have shifted over time (Table 1). In the first 2 years of the incentive metric, a CCO could earn incentive dollars by meeting the first 2 components without having to also meet or exceed a prevalence target. By the third year of the incentive metric (2018), CCOs could only earn incentive dollars if their cigarette smoking prevalence for Medicaid members aged 13 years or older was at or below 25 percent. The progressive nature of the metric (in which the required cumulative percentage increases over time from 60% to 75%) allows for stepwise implementation and achievement of the different components.

**Table 1:** Components of the CCO Cigarette Smoking Prevalence Incentive Metric, Oregon’s CCO Quality Incentive Program, 2016–2018^a^

Metric Component	2016	2017	2018
Weighted % for meeting the cessation benefit requirement (must pass); if a CCO does not meet this requirement, it cannot earn incentive dollars for this metric	40	33	25
Weighted % for reporting EHR-based prevalence data	40	33	25
Weighted % for meeting prevalence target	20	33	50
Required cumulative % to pass the metric; if the percentage is not achieved, the CCO cannot receive the incentive dollars	60	66	75

Abbreviations: CCO, coordinated care organization; EHR, electronic health record.

a Oregon had 16 CCOs in 2016, and 15 in 2017–2018.

When the cigarette smoking incentive metric was introduced in 2016, coverage of tobacco cessation benefits varied significantly across the CCOs. Embedding cessation benefit requirements into the metric specifications was intended to ensure that all Medicaid beneficiaries statewide would have access to a “benefit floor.” To meet the minimum cessation benefit requirement, each CCO must cover both counseling and FDA-approved cessation medications, as well as remove barriers to accessing the benefit (Table 2). This requirement is ascertained by a CCO survey developed by OHA staff and focuses on understanding the details of the cessation benefit each CCO offers. It is fielded annually and completed online by CCO staff who are responsible for incentive metric reporting (30).

**Table 2:** Minimum Cessation Benefit, as Required by CCO Incentive Metric, Oregon’s CCO Quality Incentive Program, 2016–2018

Counseling (Per Quit Attempt)	FDA-Approved Cessation Medications^a^ (Per Quit Attempt)	Access to Cessation Benefit
• Individual counseling, at least 4 sessions of at least 10 min each • Group counseling • Telephone counseling, multi-call benefit^b^	• Nicotine gum • Nicotine patch • Nicotine lozenge • Nicotine nasal spray • Nicotine inhaler • Bupropion SR^c^ • Varenicline	• No prior authorization needed to access nicotine gum, patches, or lozenges • No copayments, coinsurance, or deductibles • No annual or lifetime dollar limits • Must offer at least 2 quit attempts per year

Abbreviations: CCO, coordinated care organization; FDA Food and Drug Administration; SR, sustained release.

a Cessation medications must also meet minimum quantity requirements per quit attempt.

b Telephone counseling benefits can be provided by in-house CCO staff or through a contract with a quitline vendor; however, the state-funded tobacco quitline services were not counted as a CCO-covered benefit.

c Oregon also provided clarification to CCOs on how to distinguish bupropion SR for cessation from bupropion SR for depression. CCOs must include coverage for bupropion SR for cessation.

EHR-based reporting defines the cigarette smoking prevalence rate as the number of cigarette smokers among those who had an office visit with the provider during the year who have smoking and/or tobacco status recorded (Figure). The EHR-based reporting collects 3 smoking prevalence rates. The first is the rate of screening for smoking and/or tobacco use. The second is the cigarette smoking prevalence rate, and the third is the tobacco use prevalence rate. Tobacco use includes cigarettes and other tobacco products, such as snuff and chew. Rate 2, the smoking prevalence rate, is defined as the number of cigarette smokers who had an office visit with a provider during the measurement year, who have their smoking and/or tobacco use status recorded. The EHR-based prevalence rate is self-reported by CCOs and the data submission includes prevalence data for individual clinics within each CCO’s provider network. Oregon does not audit the data submissions; however, it conducts multiple layers of validation on the data to ensure accurate reporting, including 1) comparison of a CCO’s data submission to the prior year (including at the individual provider level); 2) reviewing data submissions for outliers, both across the CCOs and within a CCO’s provider network; and 3) reviewing data submissions for inconsistencies. Any of these face validity checks may result in following up with the CCO for clarification or data resubmission to ensure accuracy as part of the overall CCO incentive metric review and validation process.

**Figure:**
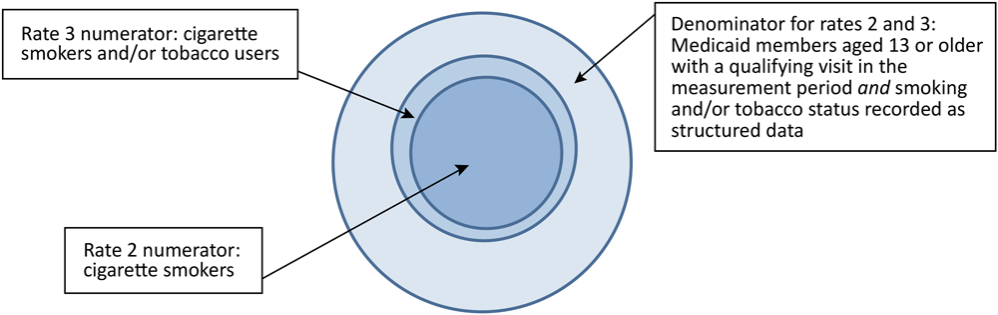
Electronic health record–based prevalence measure specifications, rate 2, Oregon’s CCO Quality Incentive Program, 2016–2018.

## Results

The comparison of CCO cessation benefits for 2014 (pre-incentive metric) and 2018 (post-incentive metric) is presented in Table 3. The cigarette smoking prevalence CCO incentive metric was first reported for calendar year 2016. In 2018, all 15 CCOs met their cessation benefit requirement, all 15 successfully reported prevalence data from EHRs, and cigarette smoking prevalence had declined in 13 CCOs since 2017 (31). Post implementation of the incentive metric, all 15 CCOs reported covering all 3 types of counseling (telephone, individual, and group) and all 7 FDA-approved medications, compared with only 14 CCOs covering all counseling types and 9 CCOs covering all medications in 2014. The number of CCOs that require prior authorization for 1 or more FDA-approved pharmacotherapies also decreased from 2014 to 2018 (from 16 CCOs requiring prior authorization for at least 1 down to 9 CCOs). No CCOs had copays or lifetime dollar limits for smoking cessation benefits.

**Table 3:** Survey of CCO Tobacco Cessation Benefits for 2014 and 2018, Oregon’s CCO Quality Incentive Program

CCO Characteristic	No.^a^ (%)	
	2014	2018
Covers all 3 types of counseling (telephone, individual, group)	14 (87)	15 (100)
Provides coverage for all 7 FDA-approved cessation medications	9 (56)	15 (100)
Requires prior authorization for at least 1 FDA-approved pharmacotherapy	16 (100)	9 (60)
Contracts with a quitline vendor for telephone counseling	10 (63)	11 (73)
Requires copayments for any cessation medications	0	0

Abbreviation: CCO, coordinated care organization; FDA, US Food and Drug Administration.

a Oregon had 16 CCOs in 2016, and 15 in 2017–2018.

In addition to benefit package improvements, Oregon also demonstrated a decline in its Medicaid cigarette smoking prevalence, as measured through EHRs between 2016 and 2018 (29.3% in 2016, 28.0% in 2017, and 26.6% in 2018) (31). Although 13 of the 15 CCOs demonstrated a decline in cigarette smoking prevalence between 2017 and 2018 on the basis of their EHR-reported prevalence data, considerable variation in cigarette smoking prevalence still exists across CCOs, ranging from 20.2% to 36.6% in 2018, based on a total of 254,111 patients (31).

Other recent statewide evaluations through CAHPS (an annual random survey of Medicaid recipients in Oregon) indicate that the CCO incentive metric has been successful in increasing provider attention to cessation. Since 2015, adult Medicaid tobacco users who reported that their doctors offered them cessation medications and other strategies to help quit has increased above baseline, with a high in 2017 and persistent gains above baseline through 2018 (Table 4) (31).

**Table 4:** Oregon Medicaid Members Who Reported Their Health Care Provider Recommended Cessation Assistance, Oregon’s CCO Quality Incentive Program, 2016–2018^a^

Characteristic	2011	2015	2016	2017	2018
Percentage of adult members who use tobacco and whose health care provider recommended medication to help quit	24.0	26.9	27.4	34.3	32.5
Percentage of adult members who use tobacco and whose health care provider recommended strategies to help quit	25.0	23.1	23.1	29.1	27.0

Abbreviations: CAHPS, Consumer Assessment of Healthcare Providers and Systems survey; CCO, coordinated care organization.

a Although CAHPS Medical Assistance with Smoking Cessation questions usually combine 3 response options (“sometimes,” “usually,” and “always”) for reporting, Oregon uses just 2 response options for the CCO measurement program (“usually” and “always”). When compared with the national Medicaid 90th percentile for these questions, measured in the same way, Oregon falls short: 32.5% compared with 60.3% and 27.0% compared with 54.1% in 2018, respectively.

### Limitations

This study has several key limitations. Although a focus on combustible cigarette use is critical, this metric did not focus on other forms of nicotine use that are also of concern: cigars, e-cigarettes, smokeless tobacco, and other tobacco and vaping products. Unmeasured confounders may exist, such as national trends that may have been the primary driver of the improvement in smoking cessation prevalence rather than Oregon’s CCO incentive metric alone. Despite this limitation, this novel outcome metric aligns public health and Medicaid to focus on a critical public health issue and improved access to evidence-based smoking cessation aids at a minimum. If the impact of this metric is smaller than reported, other benefits may still be derived from it, including holding health plans accountable to population health metrics and alignment between health plans and public health to improve outcomes with interventions spanning clinical and other sectors. Future studies could further triangulate key drivers of prevalence improvement by further investigating the use of pharmacotherapy, quitlines, counseling, and community-based interventions. The final limitation relates to generalizability. Other states and health plans may find it challenging to adopt metrics such as the Oregon smoking cessation metric. However, given the lack of viable comprehensive outcome measures on smoking cessation and the ongoing significant morbidity and mortality associated with smoking, it may be worthwhile for other systems to invest in adoption of a similar novel outcome metric.

## Implications for Public Health

Oregon’s innovative work creating an incentive metric that requires health plans to cover comprehensive evidence-based tobacco cessation benefits and be held accountable for smoking prevalence has contributed to a substantial population decrease in smoking prevalence in Oregon and improved access to evidence-based cessation aids for OHP (Medicaid) members. This success depended on coordination and alignment across Medicaid and public health coupled with the use of financial incentives and effective data monitoring. Oregon has also worked effectively across sectors such as in the case of a statewide opioid initiative (32), and increasingly this type of cross-sector collaboration is being looked to as an effective means to improve population health nationwide (8,33,35).

In addition to the statewide successes, the cigarette smoking incentive metric has created an unprecedented opportunity for local public health and health care partners to collaborate at the community level to implement effective strategies for preventing and reducing tobacco use. These efforts have led to CCOs investing time and resources in working on prevention strategies outside of the clinical setting, engaging more in their communities, and collaborating with local public health authorities.

For health plans to truly be accountable for population health outcomes, those distal outcomes need to be measured and incentivized. Reliance on process-based metrics such as the proportion of primary care providers screening for tobacco use is arguably insufficient. Similarly, if the goal is accountability to improved population health, requiring health plans simply to provide a benefit or even measure the times patients received pharmacotherapy or counseling (standard smoking cessation metric measures) is not enough. Instead, requiring plans to be accountable for the health outcome (ie, reduced smoking) by using effective data reporting, concrete guidance and contractual requirements, required minimum coverage, and financial incentive metrics to drive the change, population health improvements are achievable. However, major barriers exist to health insurers addressing prevention activities at a clinical population level or community health level, including silos between public health and Medicaid health plans, lack of funding streams to facilitate delivery of nonclinical interventions, limited coordination between clinical systems and community resources, legal barriers to Medicaid paying for nontraditional services, and concerns about upfront costs of investing in additional services and strategies.

As Oregon and other states continue to work to maximize population health with regard to tobacco use, continually promoting evidence-based strategies, regardless of the setting in which they are delivered, is important. What HERC seeks to do with the Multisector Intervention Statements is provide a menu of evidence-based options, some clinically focused and others exclusively based in the community, allowing plans to decide which of these interventions makes sense, given their priorities and community relationships and the cost–benefit ratio for each. To address the issue of tobacco in our communities, both prevention of tobacco initiation and effective treatment of tobacco use disorder are paramount to effectively reducing tobacco use prevalence.

Oregon’s innovative work in developing an incentive metric that requires CCOs to address tobacco prevalence through evidence-based strategies extending beyond the clinical setting can be a model for other states and payers seeking to effect major population health change by increasing engagement and accountability of payers and health systems. Although CCOs have improved benefits since the adoption of the incentive metric and there is some indication of clinical providers improving their practice, room for improvement still exists using the strong foundation that HERC guidance, benefit requirements, and incentive metric strategies have set.
